# The Mask of the Warrior: unraveling deep-seated health vulnerabilities in veteran identities

**DOI:** 10.3389/fsoc.2024.1389924

**Published:** 2024-10-04

**Authors:** Jan Grimell

**Affiliations:** Department of Sociology, Faculty of Social Sciences, Umeå University, Umeå, Sweden

**Keywords:** veteran, warrior, mask, *Me* (a concept for a group’s values and behaviors that constitute a particular “*Me*”), suffering, deteriorating mental health

## Abstract

As service members transition from deployment to civilian life, they are also expected to reintegrate into society. An important part of this process is to “soften up” veteran or warrior identities and open up the self for both existing and new identities, mindsets, and ways of life. Past research has shown that the warrior mindset, in particular, can have negative health implications in the long run. The mindset can be costly, not only for the individual and their loved ones, but also for the healthcare services and other agencies. This article draws from a recent interview study with 24 deployed Swedish veterans suffering from deteriorating mental health without receiving a clinical diagnosis. Purposeful sampling was conducted with the support of the medical staff at the Veterans’ Clinic at Uppsala University Hospital. Participants had been screened for posttraumatic stress disorder (PTSD) but had not received a clinical diagnosis. This constitutes a large and understudied patient group in the clinic. The medical staff selected patients based on the following criteria: deteriorating mental health, increased suffering related to PTSD symptoms, and issues related to moral issues, existential concerns, and identity. The sample included veterans from both the Swedish Armed Forces and other deploying agencies. Of the 24 interviewees, 19 were from the Swedish Armed Forces (16 men and three women), and five (four women and one man) were deployed by other agencies. The number of overseas deployments varied widely, with some interviewees having completed 1–2 deployments, while others had completed 3–8. Additionally, some interviewees had interrupted planned or ongoing deployments for various reasons. At the time of the interviews, none were serving full-time in the armed forces; all were veterans. The interviews took place during an intense wave of COVID-19 infections in Sweden in early 2022, so the majority were conducted via videoconference. The participants’ veteran identities were abductively analyzed through the mask of secrecy, the stoic mask, and the mask of denial, which are elements of the “Mask of the Warrior.” This mask functions to safeguard mission focus, to endure, to execute tasks in extremely stressful situations, and to solve operational tasks during deployments and combat operations. The analysis of the interviews suggests that certain elements in these powerful veteran identities can serve as breeding grounds for suffering later in life. The veterans in the study tended to be stoic about their deteriorating mental health, kept the suffering to themselves, and denied the harmful aspects of their deployments. Thus, the Mask of the Warrior played a counterproductive role for the individual, their friends and family, and life in the aftermath of deployments. Another implication of secrecy and denial occurred on the societal or macro and system levels due to the absence of sufficient insight, knowledge, and understanding of veterans among personnel within the healthcare system and other agencies. This made it difficult for the healthcare system, and other relevant agencies, to offer adequate care and to understand the participants’ health issues during sick leave. The perceived absence of societal and organizational rewards and benefits for veterans who risk their mental health and lives during deployment can be seen as a failing implicit work contract. This lack of recognition may lead to the corrosion of character.

## Introduction

While there are differences between military cultures, there are also shared contours that unite them, embedded within the socialization process that develops a “military *Me*”—the social integration of military values, meanings, and practices into a part of the self, conceptualized as a “military *Me”* (Grimell, 2023a). This military *Me* is socialized through and shaped by the military organization and its culture. This *Me* is shaped to function within a strict hierarchy in a military organization, governed by ranks that everyone must adhere to Hall (2012a) and Huntington (1957). Moreover, discipline is central to the functioning of military activities, and individual needs to rest, take a break, eat, or take time off for the rest of the day are excluded (Hall, 2012b; Thornborrow and Brown, 2009). Strong physical and mental capacities are essential in order to execute tasks in difficult and extremely stressful situations, which is why a lot of education, training, and activities gravitate toward ideals such as endurance, fitness, strength, and a deeply imprinted military skill set (Brotz and Wilson, 1946; French, 2005; Haaland, 2009; Woodward and Jenkings, 2011). Extremely tough and demanding achievements are rewarded and admired in a military environment and in this lies a kind of performance-driven aspect which constitutes a military *Me*. The collective is superior to the individual in military socialization and the unit takes precedence over the self (Hall, 2012a,b; Huntington, 1957). This is a prominent moral imperative during basic training or conscription, after which it remains deeply inculcated in a collectivistic military *Me* (Grimell, 2023a). In narrative identity research, this theoretical *Me* is equated with narrative identity, which represents the connection between the theory of the self and empirical research on the self (Sarbin, 1986). *Me* illustrates, on a theoretical level, a part of the self that can be empirically captured through a narrated or storied identity (Sarbin, 1986).

Loyalty to mission, to purpose, and to battle buddies are also central and elements that resonate with hierarchy, discipline and the collective. Moreover, when service members serve together in the battlefield, an (in)visible bond can develop between battle buddies. Loyalty embraces the willingness to sacrifice, even self-sacrifice (Franks, 2004; French, 2005; Grimell, 2016; Lunde, 2009; Sørensen, 2011). “Until death do us part” are words of promise said in an ecclesiastical marriage, but these can be more than just words amid war-zone deployment; it can actually be what is agreed upon between battle buddies (Grimell, 2022a).

Being able to solve combat tasks entails that service members participate, directly or indirectly, in combat and thus neutralize or kill combatants—or support this. The socialization of a military Me or identity needs to be powerful enough for service members to transgress the civilian taboo of killing other people (combatants) during operations (Bragin, 2010; French, 2005; Goldstein, 2001; Strachan, 2006; Verrips, 2006; Wilson, 2008). A warrior culture may develop, in basic training or amid deployment, that elevates mission focus, power, strength, and combat efficiency to embodied virtues. In a warrior culture, many of the contours of military culture reach extreme levels (Edström et al., 2009; Malmin, 2013). Because of elevating embodied virtues such as power and strength, deteriorating mental health is undesired and perceived as a stigmatizing weakness (Bryan and Morrow, 2011; Dickstein et al., 2010; Ganzevoort, 2008; Malmin, 2013). A stigma, in turn, is an attribute, behavior, or reputation that is socially discrediting in a particular way and can ruin, damage, or spoil an identity (Goffman, 1986). Deteriorating mental health can cause a service member to be mentally classified by the group into an unwanted, rejected stereotype rather than into an accepted, normal one. It is thus something that is hidden away or ignored by warriors for as long as possible.

Pressure, threats, intensity, and even combat, combined with isolation from the surrounding society and a time frame of many months of deployment, means that veteran identities can germinate in very special circumstances and can settle very deeply in a human being (Grimell, 2022a). It is thus challenging to dismantle or deconstruct such robust and powerful identities amid the transition to civilian life. The undoing of these profound veteran identities, however, is not met by the same military and societal commitment as the previous phases of socialization and consolidation. Just as countries sometimes want to forget their wars, they sometimes also forget the veterans deployed to conduct these wars (Agrell, 2013; Moss and Prince, 2014; Tick, 2005).

Veterans are carriers of military identities that have been cultivated to be efficient, robust, and well-functioning on the battlefield during deployment. Reconstructing such veteran identities during the transition to civilian life entails a challenge that for most veterans is a manageable process (Atuel and Castro, 2019; Blackburn, 2017; Castro and Dursun, 2019; Grimell, 2022b; Savion, 2009; Yanos, 2004). Nevertheless, deployment may change veterans’ views and perceptions of meaning, mission, and life (Coll et al., 2012; Duel et al., 2019; Grimell, 2023b; Lifton, 1992; Schok, 2009) without necessarily causing psychiatric problems, and transition may be difficult as a process but eventually resolved. Even so, the veterans have to handle a double-edged sword throughout life by having such warrior or veteran identities. Later in life, this double-edged identity can grow into a problem and hurt the individual and their surrounding network of relationships. This is because the various elements of a deeply cultivated military or warrior identity (e.g., mission focus, discipline, loyalty, self-sacrifice, endurance, strength) can manifest as inbuilt vulnerabilities in a civilian life designed to be lived in a very different way across an entire life span. While a military identity is designed to get the job done under extreme circumstances during a relatively limited temporal dimension (e.g., a 6–12-month deployment), social identities (Tajfel and Turner, 1979) that originate from civilian social life are designed in a contrasting cultural context to work across the life span. Herein lies a potential problem that has to do with whether a veteran navigates and operates life based on the powerful mindset of a warrior identity or can unite and integrate meaningful and salient veteran and civilian identities into a dialogical self (Hermans et al., 1992; Hermans, 2001a,b). If they cannot, powerful identity elements “to get the job done” during deployment can override the dialogical capacity and instead turn these elements into potential vulnerabilities later in a civilian life (Grimell, 2018, 2020).

Such powerful elements in a veteran identity can be particularly well-illustrated (as potential vulnerabilities in civilian life) by applying the concept of the Mask of the Warrior (Wertsch, 1991) to veterans who struggle with deteriorating mental health but have not received a clinical PTSD diagnosis. Thus, the purpose of this article is to present how secrecy, stoicism, and denial—elements of the Mask of the Warrior—have operated as barriers to seeking medical support in veteran identities. This mask, viewed as an abductive interpretative tool in narrative identity analysis, has proven to be an important lens for understanding both the deterioration itself and the resistance to seeking medical support among Swedish veterans.

Identity concepts for re-evaluating the well-functioning (during active service and deployment) elements of military or warrior identities in a post-deployment civilian context are scant but important for advancing the understanding of the role these identity elements can have, such as a reluctance among veterans to seek care and support for deteriorating mental health and increased suffering (Duel et al., 2019). They are also important to identify factors contributing to the depth of a medical condition that has been hampered over time by not being addressed. This warrants further investigation in medical sociology, with research specifically addressing veterans, their social networks, and the military and healthcare agencies, as well as a critical discussion about society’s responsibility for the creation of warrior identities.

### Conceptualizing a veteran identity as a Mask of the Warrior

A veteran’s identity is rooted in the military socialization that emerges from conscription, basic training, or specialist training (Huntington, 1957; Thornborrow and Brown, 2009; Verrips, 2006; Woodward and Jenkings, 2011), and continues to develop into a veteran identity during pre-deployment training, and on to the actual deployment, transition out of deployment, and life post-deployment (Atuel and Castro, 2019; Castro and Dursun, 2019; Grimell, 2022b). Different countries define veterans differently, but for the purposes of this article, a veteran is someone who has been deployed to a conflict or war zone, i.e., a deployed veteran, which is in line with a Swedish understanding. A deployed veteran has a military identity as well, which is based on military values, meanings, and practices. The military identity is embedded in a veteran identity, which leans more to the experiences before, during, and after the deployment. That being said, a military identity is considered embedded in a veteran’s identity, but the two can also be used as separate analytical concepts for organizing and interpreting interview data in various ways. The concept of a veteran is also tailored to society’s, policymakers’, and public agencies’ conceptual apparatus about those who have been deployed and the rights and compensation that such a status can provide in society (Moss and Prince, 2014). Thus, while the term ‘military’ suggests something ongoing, the term ‘veteran’ has, in Sweden, become synonymous with deployments and the politics (including society’s views) surrounding service members as they leave active duty and transition to civilian life. However, this transition does not signify the end of the military mindset, which may be implicitly suggested by words like ‘transition’ and ‘veteran.’ An ex-service member can simultaneously experience both a military identity (encompassing a military mindset, skills, behavior, etc.) and a veteran identity (related to post-deployment life, veteran policies, healthcare benefits, societal views of veterans, and conflicts they have been involved in, etc.). One does not have to exclude the other; instead, they can be understood as different facets of how organizations, communities, society, and the self interact over time from an identity perspective (Grimell, 2022a,b).

Regardless of the use of terms, a military or veteran identity is a social identity (Tajfel, 1974; Tajfel and Turner, 1979) that originates from the military social group(s) and organization(s). This is also true of other civilian social identities such as parent (i.e., family), friend (i.e., peer groups), and employee (i.e., work groups or organizations), to name but a few. These identity processes among veterans are deeply social from the very beginning to life post-deployment and include not only the military social group(s), but also how to navigate the larger social network of groups and identities within a self, seen as a dialogical self (Hermans et al., 1992; Hermans, 2001a,b; Hermans, 2002) with various narrated identity positions, depending on situation and context in life, which equate to *Me*s (Mead, 1934/2015).

The concept of the Mask of the Warrior (Wertsch, 1991), here synonymous with a *deployed narrative veteran identity*^1^ (also see Grimell, 2022a, 2023c), brings a novel model of explanation to the recognized problem of the gap, friction, conflict, or even clash between veteran and civilian identities and life worlds that can be transmitted onto the self (Grimell, 2023a; Lifton, 1992; Tick, 2005; Shay, 2002). A dominant veteran identity, seen as an extension of the military/veteran social group(s) and culture(s), can challenge the dialogical capacity of the self. The ability to integrate, dialogue, and embrace multiple (veteran and non-military) identities, perspectives, and vantage points facilitates civilian life for veterans; while the reverse can complicate life as a post-deployed veteran in a civilian social context (Grimell, 2017a,b, 2018, 2020, 2023a).

The concept of the mask was developed by Wertsch (1991) in her book *Military brats: Legacies of childhood inside the fortress* in an attempt to better understand her own, and other children’s, childhood experiences in the military context of the United States of America by interviewing grown children. Her idea of the Mask of the Warrior equates to a veteran *Me* (on theory level), which can be grasped through narrated veteran identity claims (on an empirical level).

According to Wertsch, the Mask of the Warrior is a kind of timeless constructed archetype, but not in a mythical Jungian sense. Instead, Wertsch was inspired and leaned on the dramaturgical approach of social psychologist Erving Goffman (1952), which includes concepts such as social staging and performance, inner rooms (e.g., private, at home) and outer rooms (e.g., social settings outside the home, workplace, grocery store, school) and a kind of social psychological acting that forms various social role identities of the self. In the spirit of Goffman (1952), Wertsch (1991, p. 10–11) described how the Mask of the Warrior arose through social military dramaturgy and ritual staging, which gave rise to a warrior’s acting. The dramaturgical approach should not be understood as the warrior mask becoming less real or inauthentic, or that it even becomes superficial as only a social mask or role. On the contrary, a well-played dramaturgical social role becomes the persona—the character a human being is before the world; a social character that is associated with great sacrifices, even self-sacrifice if necessary. This social identity (Tajfel, 1974; Tajfel and Turner, 1979) is deeply anchored in a wider military community, a narrower veteran group, and close-knit battle buddies.

According to Wertsch, the Mask of the Warrior has three overarching elements, traits or sub-masks with which the bearer of the mask can face the outside world, namely the secrecy mask, the stoic mask, and mask of denial. However, the mask is not only a persona for the outside world because veterans that wear this mask do not necessarily take it off when they reach the inner or private room at home. This is one key element to why the mask is so medically and sociologically interesting. Because although the military dramaturgy is played out in a specific social military context—on a military stage—elements of the mask remain on or are still activated outside of the military stage. Simply put, the mask is not something that one takes on and off as one wishes; it is not something that is controlled by the individual once it’s cultivated. The mask is better understood as an extension of the military society, culture, and veteran group onto the individual that, through the mask, becomes part of a collective that exerts great influence over the individual. The function of this collective mask is to allow service members to carry out their duty under extreme situations such as combat. The endurance of the mask can, in the long run, contribute to social issues (e.g., challenges to adjusting and interacting in various non-military social settings) and medical problems (e.g., when clinical symptoms are met with denial and secrecy, while stoically endured), which eventually will have negative effects on the individual, significant others, and society. The three modes of the mask operate in different ways.

The mask of secrecy means being silent about one’s war experiences (Wertsch, 1991, p. 39). Silent mouths are part of military life. This is linked to the gap between the military and the civilian. War and combat are neither based on nor appeal to any comprehensible logic of peace, which is why there is a reluctance to share deployment experiences with people who do not understand or have any insight or interpretation keys for this. At the same time, service members need to keep operations and missions secret for a variety of reasons, such as to maintain a military advantage over the enemy or to protect themselves from media that would create strong social reactions about what really happens in war outside a military context.

The stoic mask is one of the most central masks because a stoic approach is a king’s virtue in a warrior society and a timeless warrior ideal (Wertsch, 1991, p. 41). The stoic mask is about being able to control everything, including pain and emotions.^2^ A warrior believes in the stoic mask and the first to lose on the stoic mask are the interpersonal relationships because everyone who wears the warrior’s mask keeps everything inside and bites the bullet. No wearer of the stoic mask says what they really feel inside and there is no room for such a mask wearer to flesh out and share their emotions and pains. The suffering takes place in silence inside the stoic mask or shell. The warrior morale means that you can never give in, but just have to grit your teeth and drive on until it’s finished. This moral military imperative makes communication and dialogue with civilian society both narrow and difficult because there are moral ideals and principles in a civilian society, such as flexibility, negotiation, consensus, and compromise, that do not resonate with military values and mindsets.

The third and final sub-mask is the mask of denial, and this mask is absolutely necessary given the military activity (Wertsch, 1991, p. 44). The military reality and the craft itself mean that there is an ever-present risk of being injured or dying during education, training and, of course, focused national or international deployments. The risk of being injured or dying is managed through denial, which is assumed or considered to be the most functional way to face this existential threat. Turning the argument around: it would be unbearable if one constantly affirmed and embraced the fact that one could be injured or die during the next combat training session with live ammunition, or on the upcoming patrol missions the next day or in 2 months. One simply would not be able to go around carrying that realization all the time and the solution to it all is the mask of denial. One simply must not tear down the curtain of the myth of everyday military life and deployment. The military therefore guards a romanticized military ideal and rejects the view of the military as a dirty and spotted political reality. The defense of a romantic ideal is at the heart of the military myth. The mask of denial reduces and whittles down the seriousness of the situation and its possible implications to a harmless level. One is simply doing a job, carrying out an impersonal mission. Wertsch argues that even partners, children, and relatives need denial to cope while living with everything that surrounds their lives and if denial did not exist, identities and lives would simply crash due to the constant burden of the existential threat to life (Wertsch, 1991, p. 46).

The mask of secrecy, the stoic mask, and the mask of denial pose an imminent risk that service members and post-deployed veterans will keep everything within themselves, as experiences accumulate and grow. Wertsch sees a clear historical link between alcohol, military activities, and deployments. She suggests that alcohol facilitates and strengthens camaraderie, group solidarity and cohesion. Soldier life and alcohol have been synonymous for as long as there have been military armies. Drinking alcohol has been and still is a way of dealing with trauma and painful experiences, a kind of coping strategy that makes it easier to carry secrets and denials, and endure pain; that is, to put on the Mask of the Warrior (Wertsch, 1991, p. 49–58).

## Method

This article draws from a study that was conducted in 2022 and ethically approved by the Swedish Ethical Review Authority (reference number 2021-05410-01). The purpose of the study was, among other things, to investigate deteriorating mental health among veterans from an identity perspective, specifically in cases where the condition does not lead to a clinical PTSD diagnosis upon medical assessment. The rationale for focusing on deteriorating mental health, rather than clinical PTSD, is that this patient group in the Veterans’ Clinic at Uppsala University Hospital^3^ is nearly as large as the group diagnosed with PTSD but is understudied in a Swedish context (Grimell, 2023c). Research on this group is scarce from any perspective.

A qualitative interview method was considered particularly appropriate for the study, due to the lack of research on identity from primary sources in the Swedish context.^4^ The focus in qualitative research is on creating new understandings of subjective phenomena, such as deteriorating mental health and suffering, by focusing on people’s life-worlds, experiences, and perceptions (Clandinin and Connelly, 2000; Gorman and Clayton, 2005; Kvale and Brinkmann, 2009; Polkinghorne, 2005).

### Sample

Purposeful sampling (Merriam, 2002; Polkinghorne, 2005) was done with the support of the medical staff at the Veterans’ Clinic at Uppsala University Hospital. The participants had been screened for PTSD but were not diagnosed. The medical staff presented information about the study to patient/participants in connection with their meetings and made their selection of patients/participants based on the willingness to participate and the following selection criteria: deteriorating mental health and increased suffering related to PTSD symptoms, moral-, existential-, and identity issues. ‘Deteriorating mental health’ was based on both self-reported data (as seen in the results) and the clinicians’ assessments of the participants. Patients with a clinical diagnosis were excluded, as the study aimed to examine the understudied group of veterans with deteriorating mental health who did not meet the criteria for a clinical diagnosis.

The sample included veterans from both the Swedish Armed Forces and other deploying agencies. Beyond Wertsch’s strictly military-linked conceptualization, the understanding in this article is that the Mask of the Warrior can work well as a generic, general term for the deployed veteran identity that all interviewees in this study (regardless of deploying agency) in some sense developed. They were all trained and deployed to missions in conflict and war zones where exposure to suffering and death was evident and there was thus also a risk of being injured or killed if something went wrong. Alcohol has often been part of the coping strategy used and the interviewees not infrequently drank alcohol with buddies from deployments with whom they felt a strong sense of belonging and shared experiences. The Mask of the Warrior is thus something that can be developed and worn by anyone who has been deployed to a conflict zone, regardless of the deploying agency.

Of the 24 interviewees, 19 were from the Swedish Armed Forces (16 men and three women) and five (four women and one man) were deployed by other agencies. A total of 17 men and 7 women were included and satisfactory to reach saturation To hinder backtracking and to protect the anonymity of the interviewees, the agencies involved have not been specified (apart from the Swedish Armed Forces). Each interviewee was asked to create their own fictitious name and Table 1 presents them by their fictitious names and deploying agencies.

**Table 1:** Deploying agencies, names, and gender.

No	Participant	Gender
Deployed by Swedish armed forces		
1	Loffe	Male
2	F	Male
3	Martin	Male
4	Per	Male
5	LOLULL^1^	Male
6	Adam	Male
7	Sven	Male
8	Lars	Male
9	Roger	Male
10	Lunsare	Male
11	Fanny	Female
12	Jan	Male
13	Ifor	Male
14	Edas	Female
15	Hasse	Male
16	Patrik	Male
17	Stiffe	Male
18	Sofia	Female
19	David	Male
Deployed by other agencies		
20	Erika	Female
21	Anna	Female
22	Harald	Male
23	Gina	Female
24	Lovisa	Female

^1LOLULL had been deployed multiple times by various agencies. He was placed in this category due to his education and position as a reserve officer in the Swedish Armed Forces.^

**Figure 1:**
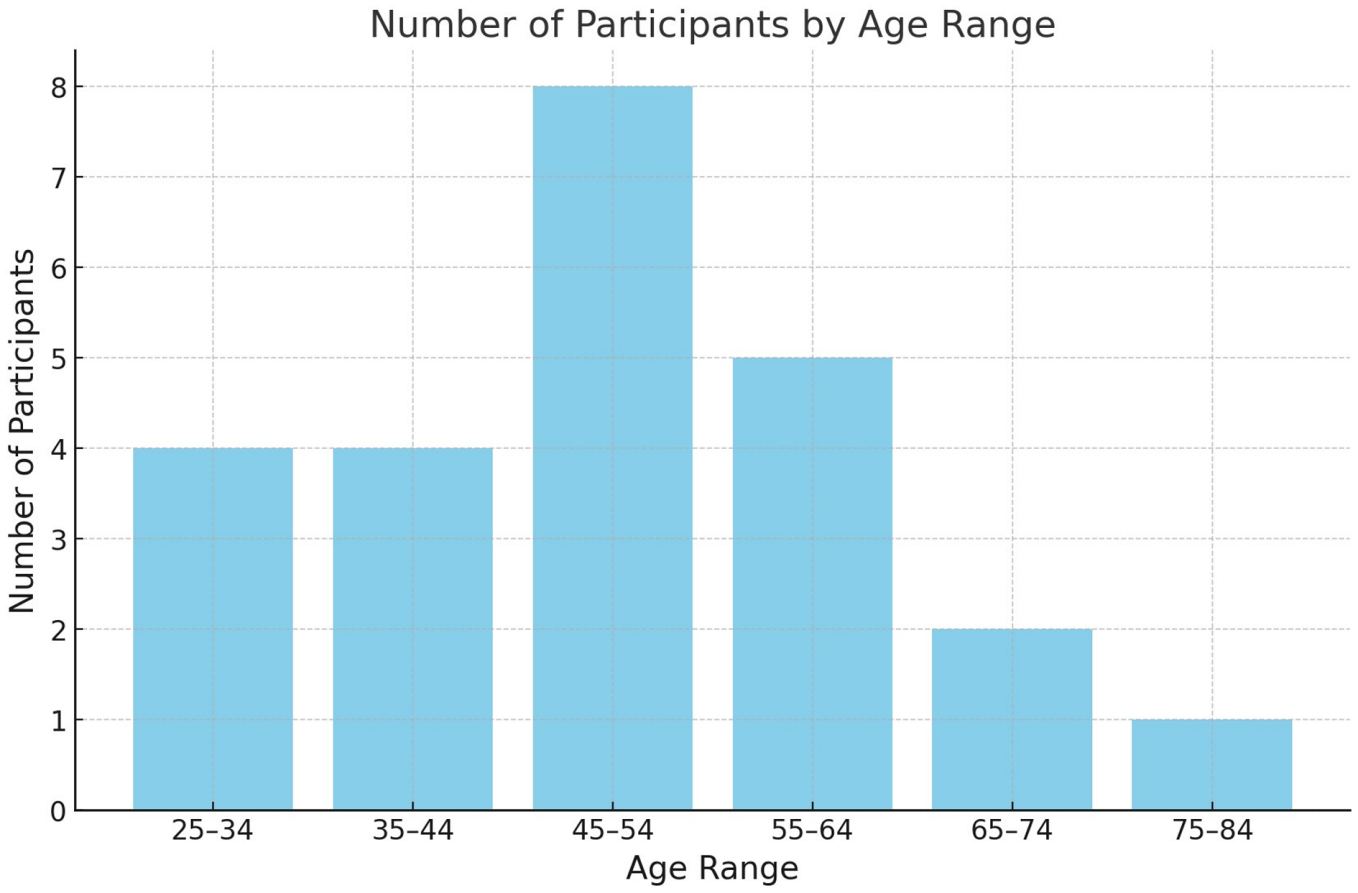
Number of participants by age range.

The age distribution of the interviewees was broad and is illustrated in Figure 1.

The army, navy, and air force—the traditional branches of defense—are represented among the interviewees, although the army and navy form the bulk of the military participants no longer serving actively. The interviewees illustrate a broad mix of different units from the aforementioned branches of the armed forces.

The number of overseas deployments varies widely among interviewees. Some have completed 1–2 overseas deployments, while others have completed 3–8 overseas deployments. There are also interviewees who interrupted planned or ongoing deployments for various reasons. The overseas deployments were carried out in different deployment areas ranging from the Middle East (e.g., Cyprus, Sinai, Lebanon), to former Yugoslavia (e.g., Macedonia, Bosnia, Kosovo), Africa (e.g., Liberia, Mali), and Afghanistan. However, the mission focus among interviewees can be said to be centered around deployments to the Middle East and what was Yugoslavia in the 1990s, and deployments to Afghanistan and Mali in the 2010s.

As for the participants deployed by other agencies, they belonged to a number of major Swedish agencies that deploy personnel to hot spots, conflict contexts, and disaster-stricken areas around the world. These interviewees have completed a large number of qualified deployments and missions and for many, but not all, this paradigm of recurring deployments is still ongoing. The deployment period among the participants extends from the 1980s until recently, and as mentioned, for some of the interviewees it is not a closed chapter either. There has been an explicit need to not disclose the agencies with which certain participants work, and I have therefore chosen to omit such information for all participants in this group. But more broadly described at group level, it can be said that the interviewees’ breadth of mission has concerned order, security, aid, care, and migration. In addition, some of these interviewees have some military training and backgrounds.

### Conceptualization of identity

The concept of identity is based on a narrative or storied understanding of identity with narrative claims on who one is (Clandinin and Connelly, 2000; Clandinin, 2013; Mishler, 1986, 2004). In other words, in the observable reality in which we live, one can approach and in an empirical sense chisel out a narrative or storied identity or character (Slocum-Bradley, 2010). Narrated identity claims can be illustrated by an interviewee being encouraged to describe who they are and how they express or make their identity in storied ways. For example, an interviewee may be asked to describe his or her military and veteran identity. This creates a narrative structure or imprint of such an identity that can then be understood through various attributes expressed in such an identity, such as units, ranks, skills, characteristics of deployments, symbols, events, episodes, experiences, and even photos associated with the military service/deployments. The terms military identity (i.e., originating from military values, meanings, and practices) and veteran identity (i.e., stemming from pre-deployment training, deployment, post-deployment, and transition into civilian life) should be regarded as analytical concepts that have proven to be fruitful tools during the course of analysis. They are neither final nor normative concepts and could be referred to in other ways (e.g., soldier, officer, specialist categories, veteran of foreign conflicts, or other meaningful names). All participants in the study were deployed veterans but not all were deployed by the armed forces, i.e., military veterans. However, although a distinction exists between military veterans and veterans deployed to conflict zones by other agencies, the Mask of the Warrior serves as an overarching generic veteran identity concept because regardless of agency, time, and place, a warrior mask was cultivated in all participants as their lives were constantly at stake while they performed their tasks amid deployments in hot spots. The deployment culture that emerged from the social interactions, tasks, threats, dangers, life-threatening incidents, combat, and so on sculpted a veteran identity that can be called the Mask of the Warrior.

### Interview design

The interview phase started during an intense wave of COVID-19 infection in Sweden in early 2022. The majority of the interviews were therefore conducted via videoconference. A number of interviews were conducted by phone due to poor internet connections, technical problems, or a lack of digital technology to enable interaction via videoconferencing. Before the interviews were conducted, informed consent had been signed and sent to the researcher. There was also an opportunity to ask questions before the interview started.

The interviews were based on a semi-structured interview design, which was formalized in an interview guide containing 25 themed interview questions that addressed a number of sub-questions regarding areas such as identity. These questions were broad, open-ended, and descriptive in nature, and had no simple answers (see Appendix A). The purpose of open-ended interview questions was to encourage interviewees to tell their own story and share their experiences (Clandinin and Connelly, 2000; Gorman and Clayton, 2005; Mishler, 1986, 2004). The open-ended question methodology creates a good opportunity for interviewees to respond to questions in their own words.

The total interview time was 29 h and 30 min, with each interview lasting roughly 1 h—some were shorter, while others were longer (for details see Appendix B).

### Analysis

The author was the sole analyzer of the interview data. The first analytical step was to manually summarize the interview narrative in a notebook after each interview. In this process, the interview was condensed down to a core narrative of one or more pages. Approximately 1½ notebooks were used in this handwritten process. This type of interview summarization into a core (life) narrative can be called a global reading, and is an approach in narrative analysis (Ganzevoort, 1998). The aim of this type of global analysis is to gain an overview of key findings and contours in the interview narrative. The notebook was not incorporated into the subsequent coding that followed, but it helped the researcher gain an overall understanding and knowledge of the 24 interviewees’ life stories during the pre-analytical phase. In the subsequent coding, all the different layers upon which the overall life stories rested were unfolded.

The next steps were transcribing the interviews, close reading, and coding of the transcribed interview material in a qualitative analysis software called Atlas.ti. This allowed the researcher to delve more systematically into the entire interview data by keeping track of all the interview codes in an easily manageable way and sorting them into different groups.

Inductive logic was used during the analysis process, which involved a movement from many individual small codes to general overall themes. The process was based on coding important and interesting individual findings in the interview material and then developing the observations into general themes or group-level categories called code families in Atlas.ti. Hypotheses and theory can be constructed and developed using the information at the group level (Kvale and Brinkmann, 2009). So, this was not a deductive process strictly based on the interview questions but rather had a freer, more inductive nature.

Not all individual codes were automatically reflected in a code family. However, the associations of a code should lead to the code family, and vice versa. Some of the individual nuance is lost in the movement from the individual to the general. At the same time, in an inductive process, individual coding needs to be sorted in some kind of way in order to create overview and order; otherwise, it becomes difficult to describe the results of the analysis in a meaningful way. The individual codes were organized into the following 13 code families (see Appendix C).

Code families 1 (*Military and veteran identity*), 2 (*Military culture/mission culture*), 3 (*Missions and experiences*), 4 (*Homecoming/transition experiences*), 5 (*Peace society and the gap to civilians*), and 6 (*Approaching deteriorating mental health in relation to the self and family*) are of particular relevance to this article. Code family 1 and 6 have a special status, while the others have an interrelated function. Finally, in order to take the analysis to an even higher level, an abductive approach was applied in which analysis and the concept of the Mask of the Warrior (Wertsch, 1991) were allowed to cross-pollinate in the results section.

All code families have been published elsewhere in a Swedish book (Grimell, 2023c) as well as in two articles that have specifically addressed, explored, and presented the moral injury dimension (Grimell, 2023d) and the experience of evil (Grimell, 2023b) among the participants.

## Results

In this results section, narrated veteran identities are approached abductively and presented through the mask of secrecy, the stoic mask, and the mask of denial. Abductively, through the components of the Warrior mask, it will be illustrated how the interviewees developed a deployed veteran identity that embraced secrecy and silence in relation to family, relatives, and others outside their inner circle. Simultaneously, they carried events, experiences, and pain in a stoic manner for as long as possible, often downplaying or even denying serious risks, events, and experiences related to deployment and life afterward. These modes of the mask often operated together.

Ranks, positions, units, specific deployment areas and other detailed information, including age, have been omitted to hinder backtracking in the presentation.

### Illustrations of the mask of secrecy operating in veteran identities

F’s deteriorating mental health debuted decades after returning home and a significant reason for this was that he had pushed aside and, in some sense, kept totally silent about his whole identity as a veteran, deployment experiences included. Upon returning home, he felt that the point of views between him and civilian friends and people in the society at peace became so contrasting that the opportunity to open up grew significantly more difficult. If he had been silent in the first phase post-deployment, F became even more silent about this veteran background across time in the social interactions, and he testified:

It started pretty soon after I got home from my deployment. I thought it would be so different when I got home. I felt that the earth gravitated around me, but coming home was like “oh well, have you been away at the grocery store?” sort of. It wasn’t that big of a deal. People tried to talk to me anyway but I wasn’t ready to talk. /…/ I chose not to talk obviously. /…/ The deployment experiences were something that I kept inside me. They were almost not allowed to come out in any way. These stayed trapped inside me.

The reason for Sven’s silence had a different angle about not burdening his family, yet a similar theme of not being able to explain the experiences to civilians was also present. Sven, who experienced a low but ever-present level of rumination due to his deployment, kept all his thoughts stemming from the deployment inside himself so as to not burden his family and testified:

I might sit and ponder a bit then and go through things with myself. But I do not want to burden the family with that. It’s nothing. So, I do not know how to explain it. I’ve never talked to my family about anything like that really. It is not something I want to burden them with. So I sort of clam up a little maybe. I have not thought like that before, but now when you ask the question, maybe it is so. […] It would never be possible to explain it.

A similar yet different theme of being overwhelmed by talking about the deployments was narrated by Harald, who had been deployed multiple times and experienced death and misery. He had to contend with a low-intensity deterioration of mental health yet did not share his experiences from deployments with loved ones and testified:

For me, it would be overwhelming to talk about it with someone who is very close. And I know my partner sometimes says she wished I could tell more, but I do not feel like I can. /.../ She has not had these experiences herself. I think it would be too heavy. It becomes too heavy to listen to it and relate to it.

Jan also experienced a low but ever-present level of rumination. He thought it was easy to internalize the soldier role but impossible to leave it behind as a post-deployed veteran in a civilian life. So the military or soldier role followed him across life. In hindsight, Jan was convinced that the return home would have been much smoother if he had talked and shared the experiences from deployment, but it wasn’t that easy, and Jan recounted:

The worst thing one could do in that situation was not to talk about it. That is, I was just talking to the wrong people. I talked, or tried to talk, to civilians who had no experience and realized that they did not understand anything. So, then I stopped talking pretty fast in my usual social circle at home. Hid the parts from deployments and then solved it as best I could.

Adam, whose deteriorating well-being debuted later in life, was silent in a similar way and kept his experiences from multiple deployments, from the first to the last homecoming, inside himself. Although the complex of experiences from the deployments affected his mental health negatively, Adam was silent and even did not tell this his partner. Adam testified:

Yes, I hid it, I would say. I guess it has to do with being strong on the outside and such. But with my partner, I may not have really talked to her either or explained or even told her how bad I really felt.

Adam’s example illustrates how the mask of secrecy and the stoic mask can cooperate in the event of deteriorating mental health. This collaboration complicates a negative health spiral in that a person is silent, displaces and hides stressing experiences, and must be strong on the outside and bite the bullet, which means that the deterioration of mental health can continue until suffering becomes too burdensome and impossible to hide.

An extreme example of silence was narrated by Fanny. Upon her return from deployment, her whole life changed quite immediately due transitional challenges, deteriorating mental health and eventually a divorce, which included the loss of her family life and her capacity to work due to sick leave. She decided to never tell anyone about her deployment and veteran identity. It became like a kind of hermetically sealed mask meant to conceal a large part of who Fanny felt she was and is, and the background that changed life from the transition to civilian life and onwards.

In Fanny’s own words:

I had decided that I would never talk to anyone, ever again, about the fact that I had been a Swedish soldier. Have had a blue beret. Never talked, had never talked about it. From [year] to [year] and I remember that I always made sure that my employers were never to tell anyone about my veteran background. I said “if you ever say I’ve been a UN soldier, I’ll quit right away. I’ll just take my paycheck and go.” But they kept the promise. They kept the promise. No one ever knew.

The last case presents how little information is shared even when the silence is eventually partly broken. Sofia, who developed an accelerating deterioration in her mental health after returning home, chose for a long time to be silent about her mental health issues until, for obvious reasons, it was no longer possible because she was on sick leave.

Sofia recounted:

It took a long time before I told [my parents] that I was on sick leave. And then I did not talk to them much about how I was feeling.

Sofia’s example showcases the limited degree of detailing the situation and really explains how she felt. It is one thing to say that one is on sick leave, but it is another thing to open up and talk about and explain why and how this relates to military life and deployment. This was hidden in the mask of secrecy.

### Illustrations of the stoic mask operating in veteran identities

The interviewees across the sample narrated how they had to develop and cultivate a capacity to perform during extreme situations and endure and control pressure, stress, pain, death, suffering, and emotions of various kinds, which resonates with a stoic mask.

David, whose deteriorating mental health debuted later in life, had built up a protective barrier and a toughness during the most demanding combat deployment.

David recounted:

I had built up some kind of protective barrier to survive mentally in some way. /…/ I think it has a price, this warrior mask or barrier. I think that in order to cope with everyday life during deployment, I had to have it, am completely convinced. Because it was a hard morning debriefing every morning. There were dead and injured all the time everywhere. […] But it [the mask] is extremely energy-intensive, both mentally and physically. I go with this inherent stress for seven, eight months and then my body does not work properly anymore. I come home and relax and then I get sick and feel bad mentally. So it has a big price, I think.

For David, molding a warrior mask was very taxing and consumed a huge amount of energy, which made him very sick when he returned home. Several years later, his mental health began to deteriorate. Lars testified to a similar toughness that he built up over the years and that resulted in a kind of mask or identity, with little or no emotions, which was another version of him. Lars recounted:

I can say, in hindsight, that I was so indoctrinated by the military that I probably did not really understand then how much military I really was. [A family member] said that “you disappeared those years. I did not really recognize you.” /.../ I did not give as many hugs. I did not let people inside of my barrier, as I did before, because I did not want to show who I was becoming.

To Lars, the identity process of becoming an experienced multi-deployed veteran changed him socially, emotionally, and behaviorally. He actively took measures to hinder his loved ones from realizing who he had become, which they obviously did anyway. This suggests that parts of him (other civilian identities) caused friction in his self, but these parts were overridden by the veteran *Me*.

Martin, whose mental health deterioration debuted during deployment, expressed a similar stoic mask that made it hard to feel anything. This was a growing problem that worried him a lot, perhaps mostly because it had not subsided several years post-deployment as a civilian. He also had a civilian job where he was exposed to death and trauma. Martin testified:

I carry many things from the deployment that I feel bad about to this day. But things that are happening now, I feel that... Maybe my backpack is full? Either I’ve learned to deal with it or I’m just pushing it in front of me. We will see in the future how it plays out, but I have a concern that I feel so little. Because it’s not just in these tragic cases that I perceive I do not feel things. I have not had any kind of relationship since the separation. I’ve dated but the emotions are simply not there, so I’ve ended them.

In addition to these accounts, the interviewees described how the veteran identities during training and deployment gravitated toward developing a mission-focused mindset designed to override fear, hunger, pain, stress, and weakness.

Adam spoke about how the military culture had socialized him to always be tough and strong in all situations:

And that’s also something that I think I’ve taken with me or carried with me. As a soldier, as a ranger, I should be tough. I cannot show weakness. I have to show strength and toughness. During deployments, I was scared several times in certain situations, but it was never something I showed. So I’ve always, outwardly, tried to be strong and solve problems and not show vulnerability or sadness.

Sofia acknowledged that her copying strategy to train and push herself beyond her limits was a role performance that had always been rewarded by officers in the armed forces. It resonated very well with the military culture and Sofia recounted:

And a lot of my behaviors, where I have not known how to handle emotions, I’ve handled it by performing or training or so, and pushing myself beyond my limits. And from all of this I got a lot of encouragement in the armed forces. These are qualities that are rewarded.

Roger, Stisse, and Hasse, all veterans from different military units in Sweden, testified to how the military culture sculpted identities that approached any given situation stoically and missioned-focused, even now, decades post-deployment as civilians.

Roger:

Actually “stop whining.” /.../ That’s what I think about the military, you survive, you learn. Hunger, sleep, they are just emotions really.

Stisse:

I’m not whining, it’s not questionable actions, it’s straightforward, I solve problems. /.../ Small problems are nothing to me, it’s almost like I get annoyed with people who have these fucking little problems.

Hasse:

I have a hard time when other people around me have a task but do not solve it. Not doing what they are supposed to.

In Stisse’s and Hasse’s accounts, the often-expressed distinction between veterans and people who do not share that cultural mindset was salient. The social categorization toward military and veteran groups was often narrated by the interviewees.

Erika, on the other hand, nuanced slightly different identity characteristics that gravitated around responsibility, duty, and standing firm in all situations:

For me, it goes back quite a lot to the fact that it is important to take responsibility and that I have a strong sense of duty and that I really value being the kind of person who stands up when difficult situations arise or become a crisis or so on.

The participants continued to describe how their veteran identities were designed around power, strength, biting the bullet, not whining, soldiering up, controlling their emotions despite extreme pressures and life-threatening situations, and keeping their emotions within themselves. These cultural testimonial accounts of veteran identity elements serve an operational definition of the stoic mask.

### Illustrations of the mask of denial operating in veteran identities

Denial was expressed in many ways among the interviewees and can be said to include a kind of generally toned-down risk of all the threats and dangers associated with deployments—a type of denial bias. The interviewees considered, only in hindsight, many years later, that they had been exposed to life-threatening and traumatic events. The perception that deployments had not been so dangerous or difficult may also have been reinforced by the armed forces, and other deploying agencies, because they did not really understand due to a perceived tendency not to report what actually happened and they did not fully know what really happened on the tactical level during the operations.

Here’s what F had to say about his experiences, where both the mask of denial on an organization level and secrecy on a personal level were illustrated:

For starters, I thought I knew everything about what was expected before I deployed. But that was just wrong. I did not really know anything. It became like a whole new world as well. [F shared traumatic events involving injuries and deaths.] I wasn’t really prepared for what a mission meant for me as a person. And during the training in Sweden before we deployed, they said that “the most dangerous thing you can do is to play floorball and travel by car.” /.../ “It’s so calm down there, it’s going to go so well, you do not have to worry.” But then it became the worst since the conflict ended. /.../ I have carried a lot inside me for almost two decades.

Loffe also had similar experiences of a small-risk deployment and recounted:

I had some information on how it was. I knew roughly what was going on and that you had a regime in [a country] and had a leader to support this regime. /.../ He had been there hiding, and there had been some problems. So, it was a small risk event when I deployed.

However, the risk turned out to be anything but small and the deployment was traumatic and eventful. Loffe and his buddies were subjected to direct threats and hostile actions as well as combat when a battle buddy was seriously wounded. In addition, there was systematic ethnic cleansing of a group of people in a nearby village. Loffe hid all this behind the mask of secrecy, as did F and the rest of the participants.

Only in hindsight did Sofia realize that she had denied the various risks of being deployed to a conflict zone. After returning home, Sofia felt that she had exposed herself to an incredible risk and this created many emotions and thoughts about responsibility, guilt, and shame in relation to herself and family.

Sofia testified:

I feel irresponsible. I feel ashamed that I have done this. I feel a guilt toward myself. But also toward my relatives. I put myself at an incredible risk when I was deployed, and I think I lived in denial about the risk of dying down there or getting injured.

In Sofia’s case and among other interviewees, a deterioration in their mental health often meant that all the masks (of secrecy, denial, and stoically enduring suffering) began to fall away and they began to reflect deeply and more intensely on the deployments, how they deceived loved ones by not talking about the risks, the potential meanings of their sufferings, and the possible implications for themselves and their close family members. For many interviewees, this was when the curtain of the myth of the masks fell from its anchors.

Jan will end this section and is representative of how the mask of denial downplayed the life-threating moments during the deployments. These experiences were dispelled to the shadowlands of his self. It was not until decades later when he talked about it during a counseling session that he realized what he had been exposed to—attacks and artillery fire to mention but a few life-threatening aspects of the deployment. This resonates with many other interviewees who many years later, in hindsight, started to realize what they had been exposed to during deployments and that they were lucky to be alive.

Jan recounted:

In an hour’s time [in therapy] when we sat there, all this had popped up and then I got all these memories back. Then I realized that it was very, very much more than I had thought it would be, so to speak, with the incidents that happened during deployment. I had probably entered some kind of denial phase. That it wasn’t so bad, what happened. But in fact, it was a tough mission for those of us who were there.

Diminishing risks and incidents during deployments were an illustration of the mask of denial in operation. The types of life-threatening events that Jan, and others, testified about had nevertheless been accumulated and stuck in their bodies and selves, no matter how much this had been denied. These pent-up experiences of threats and danger to life remained unknown yet affected the body until the curtain of myths fell and the full magnitude of episodes was retrieved through counseling or become uncovered by flashback, accident, or purposeful reflection later in life.

## Discussion

Given the results, this concluding part of the article will focus on the asymmetric military-veteran resource problem and the concept of the ‘implicit work contract’ (Sennett, 1998; Watson, 2017). It will discuss various aspects related to the warrior mask or identity, including its impact on the self, mental health, close relationships, social life, and interactions between veterans and society, such as with healthcare, social insurance agencies, the armed forces, and civilian employers.

The first and just-illustrated aspect is that there are elements in *veteran* identities (e.g., secrecy, stoically enduring mental health deterioration instead of seeking healthcare support, denial) that can have a negative impact on personal, social, and medical aspects of life post-deployment. The lingering effect of the elements within veteran identities is related to the *asymmetric military-veteran identity resource problem*, which involves a very small number of resources amid the transition from military to civilian life to make such a veteran identity work smoothly in civilian life. The socialization of military culture(s) into military and deployed veteran identities (Atuel and Castro, 2019; Duel et al., 2019; Thornborrow and Brown, 2009; Woodward and Jenkings, 2011) or *Mes* (Grimell, 2023a), and especially those warrior culture(s) and identities (Abrahamsson, 2010; Edström et al., 2009; Malmin, 2013) that may develop and manifest during conflict and war-zone deployment, have a tremendous influence on the individual service member. These socialization processes are synonymous with large and extensive military and psychiatric resources that ensure the quality of the process (e.g., psychological evaluations and selection processes, planning, protocols, boot camp instructors, working hours, buildings, equipment, materials, etc.) However, the same extensive system and organizational bulk of equal resources are not operating when a veteran leaves service as were in operation when they were socialized into the military to build a robust military and veteran identity (Duel et al., 2019). Thus, the relationship between the extent of organizational resources invested in veterans at different stages is asymmetrical: very much throughout service, and very little during the transition and reintegration into civilian society (Grimell, 2022a,b). The asymmetrical aspect is morally challenging because society, particularly through the armed forces and other agencies, has a specific responsibility to adequately support veterans. These individuals have been recruited, selected, thoroughly trained over a long period of time, and ultimately deployed to fight and protect on behalf of the state. Deployment implies that the implicit work contract—the tacit agreement between an employed individual and an employing organization about what the employee will put into the job and the rewards and benefits for which this will be exchanged (Sennett, 1998; Watson, 2017)—can be maximal, i.e., include their death. Yet even if everything goes fairly well, the individual has still been exposed to dangers and risks, and stress and frictions among partners, children, and loved ones are also part of what the individual put into the implicit contract. The rewards, benefits, and services with which the employer is expected to repay the veterans need to be on par with the implicit work contract (Watson, 2017). Otherwise, there is a risk of perceiving service and deployment as an exploitation of military personnel and veterans, which can give rise to a kind of corrosion of character (Sennett, 1998). This suggests that the veterans’, and families’, loyalties to the employer are damaged or even destroyed, which in turn can lead to a hostile or cynical attitude toward the employing organization and, in this case, society, agencies, policymakers, and civilians.

A development in the right direction is various orientation courses before, during, and after the transition from military to civilian life (see, for example, Amdur et al., 2012; English and Dale-McGrath, 2013; Fossey et al., 2019). But this is hardly commensurate with the time and resources spent on building robust military identities that become deeply embedded in the self. As presented, the mask will not simply disappear, or suspend its force, when re-integrated into civilian life only by developing and claiming various other civilian identities, such as partner, husband, wife, parent, friend, carpenter, musician, football coach, etc. The mindset of the warrior will exert a strong influence and continue to live side by side with existing and new meaningful civilian identities that develop throughout life in a (dialogical) self (Atuel and Castro, 2019; Castro and Dursun, 2019; Grimell, 2020; Lifton, 1992; Shay, 2002; Yanos, 2004). This study suggests that softening up the climate within a self needs serious, and perhaps lifelong, personal commitment to identity work, with sufficient resources provided by society. A committed approach to cultivating a dialogical climate in the self (Grimell, 2017a,b, 2018) could facilitate viewing deteriorating mental health from various non-military perspectives. This approach would help embrace and accept deteriorating mental health, reinterpret what might be seen as stigmatic weakness from a military perspective, and encourage seeking help early on (Bryan and Morrow, 2011; Duel et al., 2019; Dickstein et al., 2010), potentially changing the health trajectories for the participants at an early stage. It could have also helped to close the perceived gap between veterans and civilians entertained by secrecy and denial.

The masks of secrecy and denial can also have a particularly troublesome impact on a system level when veterans, later in life, have to deal with mental health issues due to deployments going way back in time. Through secrecy and denial, coupled with not reflecting on and clearly articulating the deeper meaning of events, healthcare and other agencies, as well as the veterans themselves, do not gain a broad, deep, and nuanced understanding of what happens during deployments and operations and how this can affect veterans. As a result, the necessary and legitimate support measures, which may extend beyond traditional medical care into other cultural venues for healing and support, are often overlooked (Bradby, 2016). For example, when it comes to the Swedish healthcare system, participants in this (Grimell, 2023c) and other Swedish studies (Grimell, 2022a; Larsson, 2019; Larsson et al., 2024) have reported experiences of being misunderstood, not believed, corrected, and not receiving adequate healthcare treatment. This also applies for veterans’ perceptions of dealing with the Swedish Social Insurance Agency (Grimell, 2022a, 2023c). A common conclusion among the Swedish researchers about veterans’ perceptions as patients within the healthcare setting is that detailed information and closer interaction between the armed forces and the healthcare system are recommended (Grimell, 2022a, 2023c; Larsson, 2019; Larsson et al., 2024). This perception of shortcomings in the healthcare context not only risks impairing adequate and quality care for veterans suffering from various mental health issues not typical for the civilian population. It also challenges the implicit work contract (Watson, 2017) about the rewards and benefits of risking mental health and lives amid deployment, followed by the risk of the corrosion of character (Sennett, 1998). As a rule, none of these perceived shortcomings and obstacles have any effects on organizations or agencies themselves. Instead, they impact the individual person in everyday life, who is forced to navigate these challenges alone against agencies and systems, often when the veterans or patients are in their worst mental condition (Grimell, 2022a, 2023c). This situation can generate and accelerate powerful feelings of societal and institutional moral betrayal (Shay, 2002). Veterans, who have been recruited, trained, and deployed by agencies on behalf of the government and have made various sacrifices (Franks, 2004; French, 2005; Grimell, 2016; Lunde, 2009; Sørensen, 2011), are then left to ‘fight’ for their rights alone. They often do so without the support of the deploying agency, as healthcare in Sweden is structured, and without being fully understood, supported, or cared for. The societal implications of secrecy and denial on a system level, plus the lack of information and close interactions between deploying agencies such as the armed forces and healthcare system, are of no benefit to either the system or the individual.

Additionally, the mask of denial can have a toxic impact on veterans’ personal meaning-making process when trying to figure out how deteriorating mental health and increased suffering originate and/or relate to deployments (Schok, 2009). Denial, combined with the prolonged onset of symptoms (1–3 decades post-deployment), makes the process of recalling experiences very challenging. This situation increases the risk that veterans may struggle to retrieve and understand how deployments contribute to their increased suffering and deteriorating mental health. It can be challenging to understand how, or if, it all links together 10–30 years after the homecoming when denial of events, incidents, risks, and exposure was the overall approach to the deployments. Testimonials suggest that it is difficult to see, address, and assess the connection between deteriorating mental health and, for instance, the low-intensity yet harmful levels of stress during an operation (Grimell, 2022a, 2023c). This can create a growing need for veterans to really find out in detail what happened during an incident or the deployment in a broader perspective. Such needs have been illustrated both in this study and in another study (Grimell, 2022a). Veterans have needed to map and grid incidents, processes, and deployments in detail to understand their own roles, the roles of others, and the broader perspective, and to connect these elements to their increased suffering and deteriorating mental health. Denial puts a smokescreen over the stakes and incidents therein (Wertsch, 1991), especially in the long run. For some, this may work well, especially if there are no mental issues at hand. For others, it can really be a complicating factor if their mental health deteriorates.

The final takeaway can be seen from a critical sociological perspective. It can, in some sense, be gratifying for both the armed forces and a society that veterans tend to be silent and stoic about their health and deployments. This may be partly related to costly reimbursement levels, war pensions, and healthcare costs in the aftermath of service (Moss and Prince, 2014), and partly to an unwillingness of the armed forces to be publicly open about what their soldiers have done during deployments in conflict areas and war zones (Agrell, 2013). For both the armed forces and politicians, it can be uncomfortable to explicitly explain in a non-technical manner what has actually been done during operations. The public may even find it offensive and upsetting to hear details of what ‘their’ soldiers did in the name of the nation, democracy, or peace during operations elsewhere (Edström et al., 2009; Nash, 2014). The formal, technical, and often laconic descriptions of the operations communicated to the public (Agrell, 2013) not only resonate with secrecy but also with a collective denial of what may be really going on (Wertsch, 1991). This denial may be exacerbated by a kind of collective public disinterest in operations abroad because civilians may just want to go about their everyday business and activities far away from the conflict or war theater (Agrell, 2013; Bélanger and Moore, 2013; Duel et al., 2019). Thus, secrecy and denial, and even stoically enduring suffering, may be elements preferred by both the generalized other (Mead, 1934/2015) within a society at peace, and by a military society (Wertsch, 1991). This can ultimately have undesirable and unfortunate consequences (e.g., insufficient care support, unnecessary suffering, insufficient support from other agencies (the armed forces included), corrosion of character, relationship problems/divorce, and so on).

## Conclusion and implications

To foster a better internal dialogue during the transition to civilian life, significantly greater support efforts should be implemented for veterans as they leave active duty. This would enhance understanding of how secrecy, stoicism, and denial can impact veterans and their social context (e.g., family, key societal support functions, and agencies). The time factor is crucial, as it may take time for a veteran to soften the warrior mask or identity and become open to new influences and ideas. Initially, practical concerns such as finding a new job, securing a salary, and having a place to live may take precedence over reflecting on how secrecy, stoicism, and denial might affect themselves and others over time.

The armed forces and policymakers need to address the asymmetric resource problem and uphold the implicit work contract, which involves high moral expectations from loyal service members (and their families) who risk their lives in conflict zones. This issue is closely tied to the moral conflicts and feelings of betrayal that veterans and families may struggle with, which can severely impact mental health. It’s important to be explicit: either adjust expectations downwards (e.g., limiting post-deployment support) or upwards (e.g., providing extensive and generous lifelong support to ex-service members), and ensure these commitments are met. Additionally, launch informative and honest campaigns to healthcare providers, social insurance agencies, and other relevant organizations to increase understanding and cultural awareness of what service members do during deployments.

While veterans diagnosed with PTSD often receive adequate medical and social support, this study highlights the challenging limbo faced by veterans who are unwell but do not receive a clinical diagnosis. Different countries have made varying levels of progress in this area; for instance, Canada has developed inclusive approaches to mental illness, such as the concept of Operational Stress Injury (OSI). Such an approach would provide the participants in this study with a recognized name for their condition, making it easier for individuals, families, employers, and social insurance agencies to understand and manage their situation.

## Data Availability

The original contributions presented in the study are included in the article/Supplementary material, further inquiries can be directed to the corresponding author.

## Footnotes

^1^In accordance with a Swedish definition by the armed forces.

^2^The stoic mask should not be confused with Stoicism, the Greek philosophical system founded by Zeno of Citium c. 300 BC and developed by him and his successors into the most influential philosophy of the Hellenistic age. While concepts from Stoicism inspired a concept like the stoic mask, it is only a superficial relationship. Stoicism is a well-developed philosophical system beyond the stoic mask.

^3^This is the only Veterans’ Clinic in Sweden.

^4^An interview study is qualitative and understanding-driven, which means that the results of the analysis cannot be transferred to a population or group of veterans. In qualitative interview research, the weight or quality of the results is not based on the number of people interviewed, although there needs to be a sufficient number of interviewees to bring out qualitative variations in the interview data. A concept such as validity can be useful in qualitative identity research (Ganzevoort, 1998; Mishler, 1991). Through a researcher’s questions about, e.g., military and veteran identities and claims thereof, qualitative research can have a high validity (ability to measure what is intended to be measured) depending on how motivated interviewees are to share their experiences. A study like this generates new knowledge and understanding, particularly concerning the implications of veteran identities among participants experiencing deteriorating mental health and increased suffering. This is essential for anyone working with or interested in deployed veterans.
