# Supplementary material for: The Mask of the Warrior: unraveling deep-seated health vulnerabilities in veteran identities
Source: Front Sociol. 2024 Oct 4;9:1389924. doi: 10.3389/fsoc.2024.1389924 (PMC11487641; doi:10.3389/fsoc.2024.1389924)
Supplement: Supplementary file 1 [file Table_1.DOCX]

**Appendix A**

**Interview guide**

**(a) Questions for exploring military/veteran identity**

1. Can you tell me in general terms about your military service (started/ended/what you did)?

2. What does the military and/or veteran identity mean to you?

3. If you think for a moment about your service/mission, can you describe the parts or periods that you feel have affected your well-being?

4. In what way have these parts/periods affected your well-being?

5. How have you experienced the return from deployment to a life at home from a well-being perspective?

**(B) Questions to explore if/how events with moral connotations affected the mood**

6. Is there any event/events that have affected your moral thoughts in a negative/bothersome/destructive way that you would like to share?

7. Would you like to describe in what way(s) this event(s) has affected your well-being?

8. Are there different parts of you (perspectives, positions) that argue/collide, please describe?

9. How has this affected your everyday life?

10. What have you done to "keep together" yourself and your well-being and make your life work (if it has worked)?

**(C) Questions to better explore an existential dimension (personal outlook on life, purpose, meaning, life questions) in the light of well-being**

11. Are there any questions about your life that have come up in light of how you feel?

12. How have your deployment (experiences) influenced your outlook on life?

13. What questions about life or life issues do you carry with you today?

14. What gives you meaning and purpose in life today?

**(D) Questions to explore the importance of relationships for well-being**

15. Could you think a little about your relationships and tell us a little about people who have been/are important to you in light of how you feel?

16. Are there any communities that mean a lot to you, and if so, please describe what they mean to you?

17. How do you feel that your trust in people has been affected by your experiences?

**(E) Questions to explore the importance of structures for well-being**

18. Which institutions and/or organizations have had an impact on your well-being?

19. What could have been done to counteract or prevent your particular bad mood?

**(F) Questions to examine how you feel today**

20. Would you like to describe how you feel today and what has the road/journey to get there looked like?

21. What thoughts and feelings do you carry with you today in relation to what you have shared?

22. How do you feel that you have been treated in different respects regarding your well-being?

**(G) Question to more deeply address narrative identity claims**

23. Who are you today, in other words, how would you describe yourself?

**(H) Closing part**

24. Are there any questions about your well-being that I haven't asked about that are important to understanding you?

25. Is there anything you want to return to that we've talked about or haven't talked about before we shut down?
