# Supplementary material for: The Mask of the Warrior: unraveling deep-seated health vulnerabilities in veteran identities
Source: Front Sociol. 2024 Oct 4;9:1389924. doi: 10.3389/fsoc.2024.1389924 (PMC11487641; doi:10.3389/fsoc.2024.1389924)
Supplement: Supplementary file 2 [file Table_2.DOCX]

**Appendix B**

Interview lengths

| **Interview** | **Lenght (hrs.min.sec.)** |
| --- | --- |
| 1 | 1.10.21 |
| 2 | 1.52.23 |
| 3 | 1.28.05 |
| 4 | 1.04.26 |
| 5 | 1.25.30 |
| 6 | 1.32.42 |
| 7 | 1.10.04 |
| 8 | 1.16.42 |
| 9 | 0.59.53 |
| 10 | 0.58.38 |
| 11 | 2.05.12 |
| 12 | 1.00.02 |
| 13 | 1.17.27 |
| 14 | 0.55.23 |
| 15 | 0.49.56 |
| 16 | 1.26.32 |
| 17 | 1.03.59 |
| 18 | 0.57.11 |
| 19 | 0.57.47 |
| 20 | 1.08.24 |
| 21 | 1.27.50 |
| 22 | 1.24.31 |
| 23 | 0.51.11 |
| 24 | 0.59.12 |
