# Supplementary material for: The Mask of the Warrior: unraveling deep-seated health vulnerabilities in veteran identities
Source: Front Sociol. 2024 Oct 4;9:1389924. doi: 10.3389/fsoc.2024.1389924 (PMC11487641; doi:10.3389/fsoc.2024.1389924)
Supplement: Supplementary file 3 [file Table_3.DOCX]

**Appendix C**

The individual codes organized into the following 13 code families.

1. Code family: Military and veteran identity.
2. Code family: Military culture/mission culture.
3. Code family: Missions and experiences.
4. Code family: Homecoming/transition experiences.
5. Code family: Peace society and the gap to civilians.
6. Code family: Approaching deteriorating mental health in relation to the self and family.
7. Code family: Different symptoms of mental illness and PTSD.
8. Code family: Moral conflicts and injuries.
9. Code family: Existential doubts and life issues/views.
10. Code family: Alcohol and cannabis to reduce symptoms and anxiety.
11. Code family: Primary care, Veterans Clinic, medical support.
12. Code family: PTG (post-traumatic growth), growth, and strategies.
13. Code family: Veteran community and camaraderie.
